# Effects of age and eccentricity on visual target detection

**DOI:** 10.3389/fnagi.2013.00101

**Published:** 2014-01-16

**Authors:** Nicole Gruber, René M. Müri, Urs P. Mosimann, Rahel Bieri, Andrea Aeschimann, Giuseppe A. Zito, Prabitha Urwyler, Thomas Nyffeler, Tobias Nef

**Affiliations:** ^1^Gerontechnology and Rehabilitation Group, University of BernBern, Switzerland; ^2^Perception and Eye Movement Laboratory, Departments of Neurology and Clinical Research, University Hospital Inselspital, University of BernBern, Switzerland; ^3^Department of Old Age Psychiatry, University Hospital of Psychiatry, University of BernBern, Switzerland; ^4^Center of Neurology and Neurorehabilitation, Luzerner KantonsspitalLuzern, Switzerland; ^5^ARTORG Center for Biomedical Engineering Research, University of BernBern, Switzerland

**Keywords:** aging, functional visual field, target detection, visual exploration behavior, visual search strategy, visual search

## Abstract

The aim of this study was to examine the effects of aging and target eccentricity on a visual search task comprising 30 images of everyday life projected into a hemisphere, realizing a ±90° visual field. The task performed binocularly allowed participants to freely move their eyes to scan images for an appearing target or distractor stimulus (presented at 10°; 30°, and 50° eccentricity). The distractor stimulus required no response, while the target stimulus required acknowledgment by pressing the response button. One hundred and seventeen healthy subjects (mean age = 49.63 years, SD = 17.40 years, age range 20–78 years) were studied. The results show that target detection performance decreases with age as well as with increasing eccentricity, especially for older subjects. Reaction time also increases with age and eccentricity, but in contrast to target detection, there is no interaction between age and eccentricity. Eye movement analysis showed that younger subjects exhibited a passive search strategy while older subjects exhibited an active search strategy probably as a compensation for their reduced peripheral detection performance.

## INTRODUCTION

Visual exploration plays a central role in everyday life. It requires a sophisticated interplay between eye movements and visual perception. The highest visual resolution is restricted to the fovea in the center of the retina (Goldberg, 1991; Henderson, 2003, 2006) and visual acuity drops rapidly toward the peripheral retina (Henderson, 2003). Humans, therefore, move their eyes rapidly to allocate the fovea on targets while exploring a visual scene (Henderson, 2003; Archibald et al., 2013). Best exploration is achieved within the central part of the visual field and objects outside the central field may be easily overlooked (Ball et al., 1988; Scialfa et al., 1994; Carrasco et al., 1995; Seiple et al., 1996; Carrasco et al., 1998; Coeckelbergh et al., 2004). In addition to target eccentricity, target detection is also influenced by the complexity of the scene, the number of targets or distractors presented, and the similarity among targets and distractors (Scialfa et al., 1998).

Aging impairs visual search, especially when exploring complex visual scenes (Scialfa et al., 1998; Madden et al., 1999), where older adults become slower and less accurate as task complexity increases (Scialfa et al., 1998; Hommel et al., 2004; Potter et al., 2012). Impairments are most pronounced when targets and distractors share similarities, or when searching for targets that are embedded within a large number of distractors (Scialfa et al., 1998). No clear age association has been found regarding target eccentricity. One study (Hommel et al., 2004) found that all age groups were affected by eccentricity effects, whereas another study (Scialfa et al., 1994) found age-related decline for peripheral target detection. Previous studies primarily investigated peripheral target detection up to about 30°. However, target detection in the periphery beyond 30° is relevant in daily activities such as driving (Goldberg, 1991; Pambakian and Kennard, 1997; Papageorgiou et al., 2007; Milleville-Pennel et al., 2010). Potter et al. (2012) suggested that age-related decline during visual exploration emerges in middle age and then progresses throughout old age. However, from a theoretical point of view, it remains unclear whether the aging effects on exploration are linear or progress, e.g., exponentially. The suggested reasons for age-dependent declines in visual search were difficulties when inhibiting irrelevant targets (Hasher et al., 1991; Commodari and Guarnera, 2008), impaired selective, or divided attention (Rabbitt, 1965; Sekuler and Ball, 1986), difficulties when spatially localizing task-relevant information (Owsley et al., 2000), and unspecific age-related slowing (Salthouse, 1993). In addition, age-dependent differences in eye movements during visual search tasks have been found, with older subjects doing more saccades than younger subjects (Scialfa et al., 1994; Scialfa and Joffe, 1997; Porter et al., 2010).

The aim of this study was to examine the effects of aging on visual search and how they are influenced by target eccentricity. Targets were embedded in images of everyday scenes to create a visual search task closer to real-life situations. We hypothesized that peripheral target detection at 10°, 30°, or 50° of eccentricity on complex backgrounds deteriorates with age. We also investigated whether age-dependent impairments for peripheral target detection progress linearly or whether performance is maintained to old age by compensation mechanisms and then deteriorates once these mechanisms fail. A further aim was to investigate age-dependent visual search strategies by analyzing eye movement exploration behavior. We hypothesized that older subjects have different search strategies compared with younger subjects.

## MATERIALS AND METHODS

### PARTICIPANTS

A naturalistic sample of 129 healthy volunteers in the Bern-Switzerland area was recruited from community centers and from the University of Bern. Care was taken to equally recruit both genders and participants equally distributed in the age range between 20 and 80 years. After a medical history that focused on past or current eye disease, participants were screened for cognitive impairment using the Montreal Cognitive Assessment (MoCA, Nasreddine et al., 2005). Twelve participants were excluded due to cognitive impairment (MoCA <26) or eye disease. The remaining 117 participants (61 male, 56 female), with a mean age of 49.6 years (SD = 17.4 years) and an age range of 20–78 years, were included. The experiment was carried out in accordance with the latest version of the Declaration of Helsinki and approved by the local ethics committee, and all participants gave written informed consent prior to the experiment.

### EXPERIMENTAL SETUP

**Figure 1:**
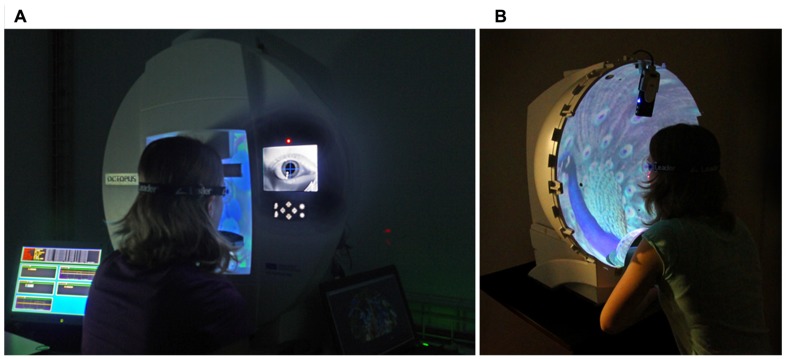
**Experimental setup**. **(A)** Hemisphere, forehead- and chin-rest. In **(B)**, the front panel is removed to illustrate the projection of the images into the cupola. The images are generated with a miniature projector positioned in the upper part of the hemisphere and reflected by a spherical mirror in the lower part (Nef et al., 2013).

A hemispheric projection screen (*d* = 60 cm, Nef et al., 2013) was used to implement a field of view of ±90° in horizontal and vertical directions. The hemisphere was positioned on a height-adjustable table (73–93 cm) with the subject seated in front of it. The height was adjusted so that the head comfortably lay on a chin- and a forehead-rest. The chin-rest was motorized and could be moved ±2 cm in horizontal and ±2.3 cm in vertical direction, while the forehead-rest moved in horizontal direction only (±2 cm). The head was positioned so that the midpoint of the left and right eye coincided with the center of the hemisphere. A miniature-projector (T25 LED, Apitec Inc., Hsinchu, Taiwan) with 800 × 600 pixel resolution was installed within the hemisphere. In combination with a spherical mirror, this setup allowed projection of images into the entire hemisphere (**Figure 1**). Further technical details of the projection are described elsewhere (Nef et al., 2013).

Participants sat in a dimmed room (0.03 cd/m^2^) in front of the hemisphere. All participants wore the same type of glasses (MediGoggle, Cambridge Research Systems LTD, Rochester, England) for optimal correction of near vision.

Eye movements were recorded with an integrated eye camera (Octopus 900, Haag Streit AG, Köniz, Switzerland) at a sampling rate of 30 Hz. A five-point-calibration was performed prior to the test to calculate the exact gaze position.

### SEARCH TASK

**Figure 2:**
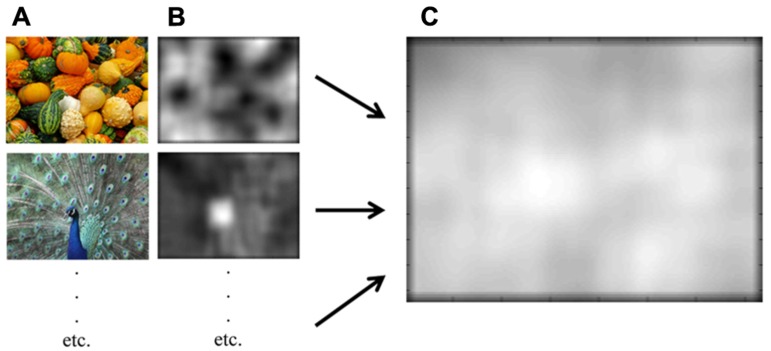
**Visual saliency analysis**. **(A)** Two example images of the functional visual field test (courtesy of Gabriele Schoenemann, Kurt Bouda/pixelio.de). **(B)** Corresponding saliency maps. **(C)** Mean saliency map over all 30 images.

**Figure 3:**
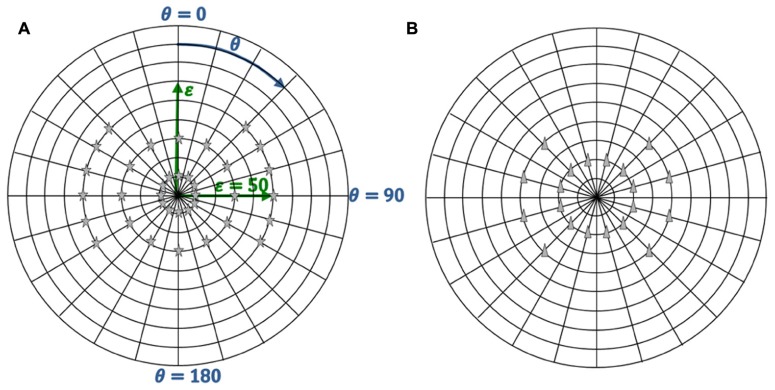
**Target and distractor positions.** **(A)** Thirty-six targets positions. **(B)** Twenty distractors positions

The visual search task was based on a functional visual field test (Muri et al., 2005). In earlier work, this test was implemented on a computer screen, resulting in a field of view of 29° × 22° (Pflugshaupt et al., 2009). With the new experimental setup, a larger field of view (±90°) could be realized. The visual search task consisted of 30 images of everyday life, such as landscapes, streets, buildings, everyday objects, edibles, or home environments (c.f. **Figure 2A**). The test was performed binocularly and participants were allowed to freely move their eyes to scan the images. Whenever participants detected an appearing small gray star (target stimulus), they had to press a response button. However, if a small gray triangle (distractor stimulus) appeared, this response should be inhibited. The design is a classical Go-NoGo Test, which has also been used by other researchers (Dwolatzky et al., 2003; Schweiger et al., 2003). Both the target and distractor were of similar size (5.21° × 8.27°), identical luminance (2.30 cd/m^2^), and identical colors. Targets appeared one at a time in one of 36 possible positions. The sequence was pseudo-randomized and 12 targets appeared at ε_H_=10°, ε_H_=30°, ε_H_=50°, respectively (**Figure 3A**). Each target position was displayed four times on four different images, resulting in 144 targets overall. Targets were visible for 2 s. Twenty distractors (**Figure 3B**) in 20 different positions were included to ensure that the search task could not be done with peripheral vision only, forcing participants to use eye movements to distinguish targets from distractors. Distractors were also shown for 2 s. Each image contained four or five targets and zero or one distractors. The time between successive targets and distractors, and the disappearance of the last target or distractor of an image and the change to the next image was pseudo-randomized between 0.5 and 2 s. The mean duration of one image presentation was 18.9 ± 2.7 s. Participants were not instructed about how many targets and distractors were shown on each image, forcing them to be constantly concentrating on detecting new targets. Prior to the experiment, a practice trial with two images (each containing four targets and one distractor) was performed. After the 30 images, the test was terminated by showing a white background.

### VISUAL SALIENCY ANALYSIS

A bottom-up visual saliency of the 30 images was analyzed to check for conspicuous image information that could bias visual attention. The algorithm of Kanan and Cottrell (2010) was used to evaluate for luminance and chromatic properties of each image and to construct a final saliency map of the 30 images of the visual search task (**Figure 2**). Concerning the central bias hypothesis, saliency values for three image regions (0–20°, 20–40°, and 40–60°) were calculated. Then, saliency quotients (specific image region/entire image) were calculated for each image. A saliency quotient significantly higher than one indicates a higher visual saliency in the specific part of the image. A similar analysis was conducted for the upper bias hypothesis (Pflugshaupt et al., 2009). Saliency values for all upper and lower image halves as well as the saliency quotient (upper/lower image halves) were calculated. In this case, a saliency quotient significantly higher than one indicates a higher visual saliency in the upper image halves.

### STATISTICAL ANALYSIS

The outcome measures of the search task included the percentage of successfully recognized targets (detection performance) and reaction time in seconds. After raw data screening, a linear broken-line regression (Robbins et al., 2006) was used to analyze the age-effects of the detection performance and the reaction time. According to the following formula, the underlying assumption is that the detection performance (respectively, the reaction time) is a function of age and would not change until a certain age (break point) after which a linear decline would occur.

(1) $$f(age)=\left\{ \begin{array}{c} {\begin{array}{c} \beta \end{array}}_{0},age<\beta_{2}\\ {\begin{array}{c} \beta \end{array}}_{0}+\beta_{1}*(age-\beta_{2}),age>\beta_{2} \end{array}\right.$$

In equation (1), β_0_ represents the baseline value, which is the performance value before the break occurs (age <β_2_). The term β_0_ + β_1_*(*x* - β_1_) describes the performance value after the break (age >β_2_), with β_1_ representing the slope of the linear change, which represents the annual change. β_2_ represents the age at the break point (age when change starts). The values for β_0_, β_1_, and β_2_ were determined for the detection performance and the reaction time for targets of all three eccentricities with the sum of least squares approach using the MATLAB calculation software (The Mathworks Inc.). The 90% confidence intervals and the *r*^2^ value as a measure of goodness-of-fit were calculated with the same software.

In addition to the linear broken-line regression, a polynomial regression of degree 3 was used to analyze the age-effects on detection performance and on reaction time:

(2) $$f\left(age\right)=\alpha_{0}+\alpha_{1}*age+\alpha_{2}*age^{2}+\alpha_{3}*age^{3}$$

Gaze positions were analyzed for each frame using the MATLAB software (The Mathworks Inc.) and were grouped into four visual field areas: 0–20°, 20–40°, 40–60°, and more than 60°. After the raw data screening, a linear regression *f*(age) = γ_0_ + γ_1_ * age | 20 < age < 80 was used to analyze the effect of age on gaze position during the visual search task using the same software. γ_0_ represents the baseline value, which is the percentage of gaze position in a specific visual field area, and γ_1_ is the slope of the linear change, which represents the annual change. The values for γ_0_ and γ_1_ were determined for the gaze position of the four visual field areas using the sum of least squares approach. The 90% confidence intervals and the *r*^2^ values as a measure of goodness-of-fit were calculated with the same software. In addition, we analyzed the distance between gaze position and target position at target onset. The mean and the variance values were calculated for young (20–40 years) and older (60–80 years) subjects.

Correlations between target detection performance and gaze position, reaction time and gaze position, and age and gaze position were calculated with Pearson’s correlation for parametric data and with Spearman’s correlation for non-parametric data.

The visual saliency analysis revealed the mean value and its standard error (SE) of all images for the three image regions (0–20°, 20–40°, 40–60°) vs. the entire image and for the upper vs. the lower image. The mean values were tested for statistical significance against a value of 1 with one-sample *t* test (Pflugshaupt et al., 2009).

In general, a *p* < 0.05 was considered statistically significant. The reported *p*-values are one-tailed for the correlation analysis and target distance analysis (Mann–Whitney test), and two-tailed (*t*-test) for the visual saliency analysis.

## RESULTS

### SEARCH PERFORMANCE

**Figure 4:**
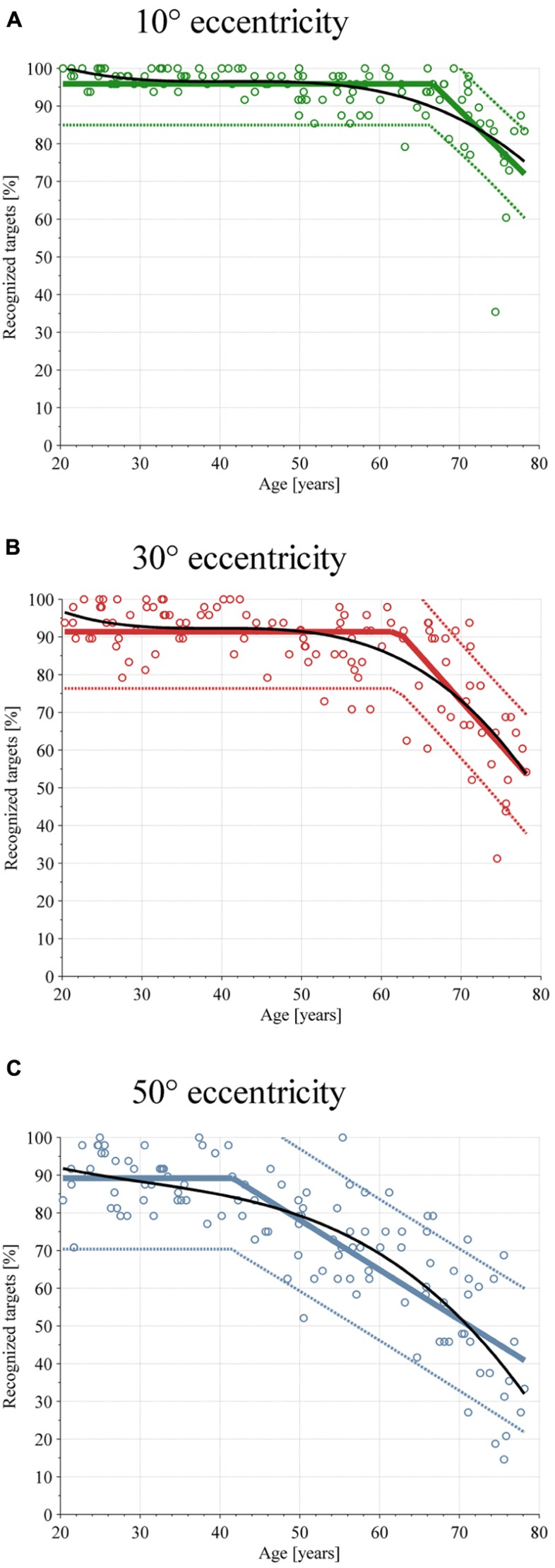
**Scatterplot of target detection performance depending on age**. Each subjects’ performance is shown with circles, linear broken-line regression with colored solid lines, and the 90% confidence interval is shown with dotted lines and the polynomial regression as a solid black line. **(A)** Performance at ε_H_ = 10°. **(B)** Performance at ε_H_ = 30°. **(C)** Performance at ε_H_ = 50°.

In **Figure 4**, the percentage of recognized targets is shown in three separate plots for targets presented at 10°, 30°, and 50° eccentricity. Each data point corresponds to one participant. Therefore, each plot contains 117 data points. The linear broken-line regression and the corresponding 90% confidence intervals are represented in the figures as well. For targets presented at 10° eccentricity, the baseline recognition rate β_0_ is 95.88%, which is the value of the linear broken-line regression for the age 20–66.66 years. After the age 66.66 years (break point β_2_), the target recognition performance decreases by -2.07% per year (slope β_2_). The values β_0_, β_1_, β_2_, and the *r*^2^ values for the three investigated eccentricities are presented in **Table 1**. **Figure 4** also shows the polynomial regression analysis for the recognized targets. The values of the polynomial coefficients and the *r*^2^ values are shown in **Table 2**.

**Table 1:** Values of the linear broken-line regression analysis for the target detection performance and the reaction time.

	β_0_	β_1_	β_2_ (years)	*r*^2^
**Target detection**				
• ε_H_ = 10°	95.88%	-2.07%/year	66.66	0.46
• ε_H_ = 30°	91.39%	-2.37%/year	62.23	0.59
• ε_H_ = 50°	89.20%	-1.32%/year	41.60	0.69
**Reaction time**				
• ε_H_ = 10°	0.62 s	0.01 s/year	42.34	0.33
• ε_H_ = 30°	0.74 s	0.01 s/year	41.06	0.33
• ε_H_ = 50°	0.88 s	0.01 s/year	41.06	0.37

ε_H_, *Eccentricity of the target; β _*0*_, baseline value; β _*1*_, slope (annual change);*

β_*2*_, break point (age when change starts).

**Table 2:** Values of the polynomial regression analysis for the target detection performance and the reaction time.

	α_0_	α_1_	α_2_	α_3_	*r*^2^
**Target detection**					
• ε_H_ = 10°	100.25%	-2.18	0.05	-4.28 × 10^-4^	0.46
• ε_H_ = 30°	96.50%	-2.96	0.08	-6.48 × 10^-4^	0.61
• ε_H_ = 50°	91.74%	-1.40	0.04	-3.85 × 10^-4^	0.71
**Reaction time**				
• ε_H_ = 10°	0.61 s	5.57 × 10^-3^	-1.71 × 10^-4^	2.15 × 10^-6^	0.33
• ε_H_ = 30°	0.73 s	-1.05 × 10^-4^	-1.15 × 10^-5^	8.38 × 10^-7^	0.33
• ε_H_ = 50°	0.90 s	-2.30 × 10^-2^	4.98 × 10^-4^	-2.50 × 10^-6^	0.36

ε_H_, *Eccentricity of the target; α _*0*_, α _*1*_, α _*2*_, α _*3*_, are the polynomial coefficients.*

**Figure 5:**
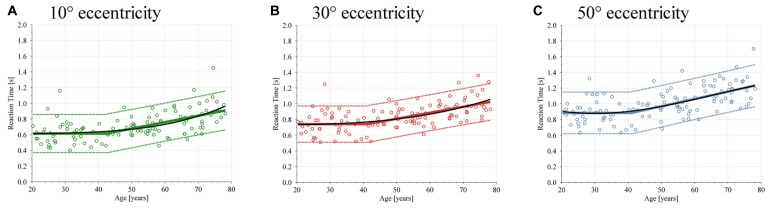
**Scatterplot of the reaction time depending on age**. Each subjects’ performance is shown with circles, linear broken-line regression with colored solid lines, and the 90% confidence interval is shown with dotted lines and the polynomial regression as a solid black line. **(A)** Reaction time at ε_H_ = 10°. **(B)** Reaction time at ε_H_ = 30°. **(C)** Reaction time at ε_H_ = 50°.

The reaction time, defined as the time between the appearance of a target and when the subject presses the button, is shown in **Figure 5** for targets presented at 10°, 30°, and 50° eccentricity. Also here, each graph contains 117 data points, the linear broken-line regression and the 90% confidence interval, and the polynomial regression. The baseline values of the linear broken-line regression of the reaction time are 0.62, 0.74, and 0.88 s for 10°, 30°, and 50° eccentricities. Starting at the ages 42.34 years (ε_H_ = 10°), 41.06 years (ε_H_ = 30°), and 41.06 years (ε_H_ = 50°), the values of the linear broken-line regression increase by 0.01 s per year for all three eccentricities. The regression coefficients and *r*^2^ values for the linear broken-line regression are represented in **Table 1** and for the polynomial regression in **Table 2**.

### GAZE POSITIONS

**Figure 6:**
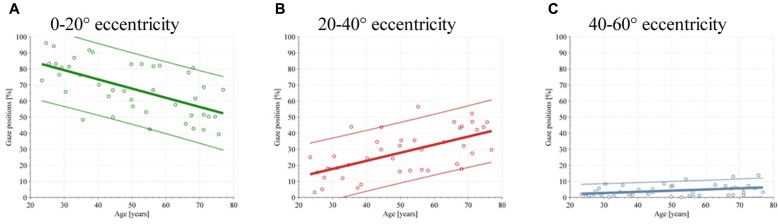
**Scatterplot of the gaze position during the test depending on age**. Each subjects’ performance is shown with circles, linear regression with colored solid lines, and the 90% confidence interval is shown with dotted lines. **(A)** Gaze position between 0 and 20°. **(B)** Gaze position between 20 and 40°. **(C)** Gaze position between 40 and 60°.

**Figure 6** shows an age-dependent effect in visual exploration behavior during the search task. Young subjects focus more on the central 20° compared with older subjects. By contrast, the older the subject, the more gaze positions are within 20–40° of the visual field. No increase or decrease during aging was found for gaze positions over 60°. The *r*^2^ values are represented in **Table 3**.

**Table 3:** Values of the linear regression analysis for the gaze position.

	*γ*_0_ (%)	*γ*_1_ (%/year)	*r* ^2^
**Gaze position**			
• 0–20°	84.95	-0.57	0.36
• 20–40°	12.73	0.50	0.38
• 40–60°	2.03	0.07	0.12

γ_0_, *Baseline value; γ_1_, slope (annual change)*.

At target onset, the mean distance between gaze position and target is significantly higher for older subjects as compared to younger subjects (young: mean = 28.76°, SD = 11.34°; old: mean = 41.60°, SD = 13.20°, *p* = 0.005). Furthermore, older subjects have a significant higher variance in target distance compared to younger subjects (young: mean = 658.05°, SD = 489.27°; old: mean = 932.13°, SD = 545.54°, *p* = 0.026).

### CORRELATIONS BETWEEN TARGET DETECTION PERFORMANCE REACTION TIME/AGE AND GAZE POSITIONS

**Table 4** shows correlations between target detection performance and gaze positions, and reaction time and gaze positions. Both target detection performance and reaction time are significantly correlated with gaze positions up to 60°, after which no significant correlation occurs. Target detection performance is positively correlated with gaze position between 0 and 20°, and negatively correlated with gaze positions between 20 and 40°, and 40 and 60°. On the other side, the reaction time is negatively correlated with gaze positions between 0 and 20°, and positively correlated with gaze positions between 20 and 40°, and 40 and 60°.

**Table 4:** Correlations between target detection performance/reaction time/age and gaze position.

Gaze position	0–20°		20–40°		40–60°	
	*r*	*p*	*r*	*p*	*r*	*p*
**Target detection**						
• Total	0.567	<0.001	-0.555	<0.001	-0.323	0.019
• 10°	0.559	<0.001	-0.499	<0.001	-0.298	0.028
• 30°	0.499	<0.001	-0.569	<0.001	-0.357	0.010
• 50°	0.559	<0.001	-0.567	<0.001	-0.313	0.022
**Reaction time**						
• Total	-0.486	0.001	0.467	0.001	0.400	0.002
• 10°	-0.512	<0.001	0.488	0.001	0.561	<0.001
• 30°	-0.460	0.001	0.432	0.002	0.513	<0.001
• 50°	-0.393	0.005	0.373	0.008	0.379	0.007
• Age	-0.633	<0.001	0.639	<0.001	0.360	0.010

*r*, Correlation coefficient.

**Table 4** also shows a significant negative correlation between age and gaze position between 0 and 20°, and a significant positive correlation between age and gaze positions 20 and 40°, and 40 and 60°. There is no correlation between age and gaze position over 60°.

### VISUAL SALIENCY

**Table 5** shows that, over all images, the visual saliency in 0–20° image regions did not significantly differ from that of the entire image. This is also true for the 40–60° image region. However, the visual saliency for the image region between 20 and 40° differs from that of the entire image. In addition, the visual saliency of upper image halves was similar to that of lower image halves. **Figure 2C** shows the visual saliency map over all images.

**Table 5:** Comparison of visual saliency in three image regions (0–20°, 20–40°, 40–60°) with the entire image as well as the comparison of the upper image halves with the lower image halves (expressed as saliency quotient).

Saliency quotient	Mean	SE	Test value	*T*	df	p
0–20°/entire image	1.034	0.024	1	1.435	29	0.162
20–40°/entire image	1.024	0.011	1	2.073	29	0.047
40–60°/entire image	1.011	0.006	1	1.786	29	0.084
Upper/lower image	0.975	0.018	1	-1.390	29	0.175

*SE, Standard error. Analyses are based on all images (*N* = 30) of the functional visual field test.*

## DISCUSSION

The primary aim of the study was to investigate the visual search task for targets appearing in the center and in the periphery in healthy subjects over a large age range and to investigate their visual exploration behaviors. The results show that peripheral target detection performance at 50° starts to decrease at age 41, whereas the performance at 30° eccentricity remains stable until age 62, and the detection of targets at 10° eccentricity until age 66. In addition to the linear broken-line regression model, the polynomial regression model also shows a decrease in target detection performance with increasing age for all three target eccentricities, especially for peripheral targets. In contrast, the reaction time starts to increase at the age of 41 for all eccentricities. The polynomial regression model confirms this result in that it shows a similar increase in reaction time for all three eccentricities. The values of *r*^2^ of both regression models have been used as a measure of goodness-of-fit (**Tables 1, 2**). Using the criteria of Cohen (1992) all *r*^2^ values reveal a strong effect size (*r*^2^ ≥ 0.33). The results of the visual exploration behavior demonstrate differences in gaze positions during the test. The older the subject, the lesser gaze positions within 0–20° of the visual field. The values of *r*^2^ of the linear regression analysis show strong effect sizes for gaze position between 0 and 20° and 20 and 40° (*r*^2^ ≥ 0.36). However, the effect size for the gaze position between 40 and 60° is low (*r*^2^ = 0.12). As a novelty, we included more peripheral eccentricities and embedded targets and distractors into complex backgrounds. Both hypotheses, a decreased peripheral target detection performance and an altered visual search strategy for older subjects, were confirmed.

Target detection performance showed age- as well as eccentricity-dependence. Eccentricity-dependence was also found in several studies about visual search performance. In all studies, the greater the target eccentricity, the less accurate the observers’ performance, irrespective of age (Ball et al., 1988; Scialfa et al., 1994; Carrasco et al., 1995; Seiple et al., 1996; Carrasco et al., 1998; Coeckelbergh et al., 2004). We believe that eccentricity-related changes in target detection performance can be caused by visual or attentional accounts or by a combination of these two accounts. These two reasons for eccentricity-related changes in target detection performance have also been discussed controversially in literature. While some researchers argue for a dominant visual account (spatial resolution) of eccentricity effects (Carrasco and Chang, 1995; Carrasco and Frieder, 1997), others support a dominant attentional account (Ikeda and Takeuchi, 1975; Ball et al., 1988; Graves et al., 1993).

The age effects of reduced target detection performance are very consistent in our data and were also observed by others (Ball et al., 1988; Sekuler et al., 2000; Coeckelbergh et al., 2004; Hernandez Luna, 2010). Several studies showed that the reported age effect is task dependent (Madden et al., 1999; Hommel et al., 2004). When the task is not demanding enough, for example if the target is presented with high contrast to the background and there is a corresponding “pop-out” effect, older adults’ performance seems to be quite robust. In more complex tasks that require more cognitive or visual effort, older adults perform worse than younger adults. However, Owsley et al. (2000) demonstrated a somewhat opposite result. In her experiment, older subjects already showed spatial localization problems in very simple tasks compared with younger subjects, especially with increasing eccentricity. Various hypotheses for age-related decreases in visual search performance have been discussed in literature. Plude and Hoyer (1985) proposed the spatial localization hypothesis, claiming that age decrements in selective attention are due to a decline in the ability to locate task-relevant information in the visual field. On the other hand, the perceptual window hypothesis describes that older adults see fewer elements at once when fixating a display, thus necessitating multiple fixations to locate a target (Cerella and Poon, 1981). In addition, the useful field of view hypothesis suggests that dual tasks, conspicuity of the target, and brief stimulus durations can shrink the UFOV with age (Ball et al., 1990). However, our results concerning visual exploration behavior show variations in visual search strategies that correlate with aging. Young subjects use a passive search strategy and focus in the center of the image until a new target/distractor appears, which captures their attention, whereas older subjects have a more active search strategy and search actively for targets. Correlation analysis between visual search strategy and target detection performance showed that the passive search strategy outperforms the active search strategy. This is also in accordance with other studies that showed that a passive search is more efficient than an active search, especially in complex visual tasks (Boot et al., 2006; Smilek et al., 2006a, 2006b; Becic et al., 2007, 2008). We believe that older subjects try to compensate for a restricted peripheral attention by using an active search strategy which worsens target detection performance. This is also supported by the higher variance in the distance between gaze position and target at target onset for older compared to younger subjects. The higher variance is a consequence of the more active search strategy of older subjects (c.f. **Figure 6**) which can contribute in parts to the lack of performance. Nevertheless, it has been shown that young and old active searchers can improve their visual search performance by adopting a passive search strategy (Boot et al., 2006; Becic et al., 2008). In contrast to our results, Acik et al. (2010) found that older subjects with more explorative behavior displayed better performance in their recognition task. This could be the result of a different study design. In the study conducted by Acik et al. (2010), participants explored images for 5 s. Afterward, they were shown a circular image patch and were asked whether this patch was part of the image that had just been shown. This patch-recognition task requires visual memory rather than target detection performance. In our study, visual memory was not measured as the background image was not important. Participants just had to focus on the appearance of a new target. In this case, the best strategy was to wait until a target or a distractor appeared.

The interaction “age × eccentricity” has been discussed controversially in literature. While some researchers detected no interaction in target detection performance (Seiple et al., 1996; Scialfa and Joffe, 1997; Hernandez Luna, 2010), others showed an interaction in the performance of target detection (Sekuler and Ball, 1986; Scialfa et al., 1987; Owsley et al., 2000; Coeckelbergh et al., 2004). Our results revealed a very clear “age × eccentricity” interaction for target detection performance. The discrepancy could be caused by different experimental designs (e.g., different eccentricities tested, dual tasks vs. single task, or more importantly, free-viewing vs. fixed-viewing condition) or statistical analysis (e.g., log transformation vs. linear analysis) (Coeckelbergh et al., 2004). Coeckelbergh et al. (2004), for example, found an inverted “age × eccentricity” interaction, meaning that eccentricity effects were smaller among older subjects compared to younger subjects, in contrast to a regular “age × eccentricity” interaction found in ours as well as in other studies (Sekuler and Ball, 1986; Owsley et al., 2000). They explained the different kind of interaction by the log-transformation of the data. However, with linear data analysis, a regular “age × eccentricity” interaction was observed (Coeckelbergh et al., 2004). In addition, Sekuler and Ball (1986) showed an “age × eccentricity” interaction, but this effect was found for errors in target localization rather than failure of detection. In contrast, Sekuler et al. (2000) demonstrated such an interaction only in the easier focused-attention condition, but not in the more demanding divided-attention condition.

Similar to the target detection performance, the reaction time also showed age- as well as eccentricity-dependence. Several studies have found an eccentricity-dependence on reaction time during visual search tasks. In all studies, and irrespective of age, the greater the target eccentricity, the slower the observer’s performance was (Scialfa et al., 1994; Carrasco et al., 1995; Carrasco et al., 1998; Wolfe et al., 1998; Hommel et al., 2004). This is in accordance with our results.

Furthermore, the age effects of longer reaction times with increasing age are consistent and were demonstrated in literature (Plude and Doussard-Roosevelt, 1989; Scialfa et al., 1998; Hommel et al., 2004; Tun and Lachman, 2008; Potter et al., 2012; van de Laar et al., 2012). Similar to target detection performance, this age-dependent effect on reaction time is task dependent, resulting in longer reaction times as task complexity increases (Plude and Doussard-Roosevelt, 1989; Scialfa and Joffe, 1997; Scialfa et al., 1998; Hommel et al., 2004; Potter et al., 2012).

The results for reaction time did not reveal an “age × eccentricity” interaction, which means that – in contrast to target detection performance – target eccentricity has no influence on the age at the change point, after which an increase in reaction time occurs. The interaction “age × eccentricity” has been discussed controversially in literature. While Hommel et al. (2004) found no interaction, others found such an interaction (Scialfa et al., 1987, 1994; Plude and Doussard-Roosevelt, 1989; Scialfa and Joffe, 1997). Two possible reasons might explain a lack of “age × eccentricity” interaction for reaction time in our study. First, in contrast to target detection, we only measured and analyzed reaction time for correct answers and did not account for missed targets. The fact that older subjects miss more targets than younger subjects (especially in the periphery) could be an explanation for the lack of an “age × eccentricity” interaction for the reaction time. A second reason could be the test instruction, which was to press the button after recognizing the appeared stimuli as a target. We did not specifically instruct the subjects to press as fast as possible, and therefore we did not measure reaction times *per se*. However, the hypothesis that older subjects stall a decision (whether target or distractor) to avoid false positives can be refuted. Our data show more false positive answers with increasing age. We therefore believe that the longer reaction time is not caused by the decision “target or distractor,” but rather by initiating and performing the correct answer. This is consistent with the results of van de Laar et al. (2012), who segmented the reaction time into pre-selection time (time interval before motor cortex activation), pre-motor time (time interval to build up and activate the motor units that control the correct response), and motor time (time interval from EMG activity to overt response) and found that pre-motor and motor times were longer for older adults compared to younger adults, whereas pre-selection time was similar. This indicates that longer reaction times for older adults are caused by a longer time window to initiate and implement the correct response.

The visual search analysis revealed no visual saliency in image regions between 0 and 20° and 40 and 60°. However, visual saliency in the image region between 20 and 40° is slightly higher than that of the entire image. Acik et al. (2010) showed that younger subjects are more susceptible to bottom-up mechanisms compared to older subjects. According to their study, we would expect more gaze positions in image regions between 20 and 40° for younger subjects. Nevertheless, our results did not confirm this. The saliency quotient for 20 and 40°/entire image is very close to 1, indicating a very small deviation from 1. We believe that the significant value is caused by the small SE and therefore, this image region does not lead younger subjects to pay attention to this region.

In sum, the present study showed age- as well as eccentricity-effects for both target detection performance and reaction times, and an “age × eccentricity” effect for target detection performance. To the authors’ knowledge, this is the first study to analyze a visual search task with targets appearing up to 50° in the periphery. Furthermore, we found an age-dependent effect on the visual search strategy, where the passive visual search strategy of young subjects outperforms the active search strategy of older subjects.

Finally, two limitations of the present study need to be mentioned. First, no head movements were allowed. This is in contrast to activities of daily living where both head and eye movements to peripheral targets occur. However, head-fixed has the advantage in that all participants had a constant viewing distance. Second, the test duration of 11 min was relatively long. This could have led to concentration problems. For this reason, we checked the data and did not observe a performance decrease with ongoing test duration.

Future research could include testing subjects with visual field defects to examine compensational strategies or testing the visual search under different light conditions to account for variations in light conditions that occur during activities of daily living.

## Conflict of Interest Statement

The authors declare that the research was conducted in the absence of any commercial or financial relationships that could be construed as a potential conflict of interest.

## AUTHOR CONTRIBUTIONS

Research questions and study design were formulated by all authors. The measurements were carried out by Nicole Gruber and Andrea Aeschimann. All authors contributed to the data analysis and to the manuscript writing.

## References

[B1] Acik A. Sarwary A. Schultze-Kraft R. Onat S. Konig P. (2010). Developmental changes in natural viewing behavior: bottom-up and top-down differences between children, young adults and older adults. *Front. Psychol.* 1 207 10.3389/fpsyg.2010.00207. PMC3153813 21833263

[B2] Archibald N. K. Hutton S. B. Clarke M. P. Mosimann U. P. Burn D. J. (2013). Visual exploration in Parkinson’s disease and Parkinson’s disease dementia. *Brain* 136 739–750 10.1093/brain/awt005. 23436502

[B3] Ball K. K. Beard B. L. Roenker D. L. Miller R. L. Griggs D. S. (1988). Age and visual search: expanding the useful field of view. *J. Opt. Soc. Am. A* 5 2210–2219 3230491 10.1364/josaa.5.002210

[B4] Ball K. K. Roenker D. L. Bruni J. R. (1990). “Developmental changes in attention and visual search throughout adulthood ,” in *The Development of Attention: Research and Theory* ed. Enns J. T. (Amsterdam: Elsevier Science Publishers B.V.) 489–508 10.1016/S0166-4115(08)60472-0

[B5] Becic E. Boot W. R. Kramer A. F. (2008). Training older adults to search more effectively: scanning strategy and visual search in dynamic displays. *Psychol. Aging* 23 461–466 10.1037/0882-7974.23.2.461 18573020

[B6] Becic E. Kramer A. F. Boot W. R. (2007). Age-related differences in visual search in dynamic displays. *Psychol. Aging* 22 67–74 10.1037/0882-7974.22.1.67 17385984

[B7] Boot W. R. Kramer A. F. Becic E. Wiegmann D. A. Kubose T. (2006). Detecting transient changes in dynamic displays: the more you look, the less you see. *Hum. Factors* 48 759–773 10.1518/001872006779166424 17240723

[B8] Carrasco M. Chang I. (1995). The interaction of objective and subjective organizations in a localization search task. *Percept. Psychophys.* 57 1134–1150 10.3758/BF03208370 8539089

[B9] Carrasco M. Evert D. L. Chang I. Katz S. M. (1995). The eccentricity effect: target eccentricity affects performance on conjunction searches. *Percept. Psychophys.* 57 1241–1261 10.3758/bf03208380 8539099

[B10] Carrasco M. Frieder K. S. (1997). Cortical magnification neutralizes the eccentricity effect in visual search. *Vision Res.* 37 63–82 10.1016/S0042-6989(96)00102-2 9068831

[B11] Carrasco M. McLean T. L. Katz S. M. Frieder K. S. (1998). Feature asymmetries in visual search: effects of display duration, target eccentricity, orientation and spatial frequency. *Vision Res.* 38 347–374 10.1016/S0042-6989(97)00152-1 9536360

[B12] Cerella J. Poon L. (1981). “Age and parafoveal sensitivity ” *Paper presented at the meeting of the Gerontological Society of America* Toronto

[B13] Coeckelbergh T. R. Cornelissen F. W. Brouwer W. H. Kooijman A. C. (2004). Age-related changes in the functional visual field: further evidence for an inverse age x eccentricity effect. *J. Gerontol. B Psychol. Sci. Soc. Sci.* 59 11–18 10.1093/geronb/59.1.P11 14722334

[B14] Cohen J. (1992). A power primer. *Psychol. Bull.* 112 155–159 10.1037/0033-2909.112.1.155 19565683

[B15] Commodari E. Guarnera M. (2008). Attention and aging. *Aging Clin. Exp. Res.* 20 578–584 19179843 10.1007/BF03324887

[B16] Dwolatzky T. Whitehead V. Doniger G. M. Simon E. S. Schweiger A. Jaffe D.(2003). Validity of a novel computerized cognitive battery for mild cognitive impairment. *BMC Geriatr.* 3 4 10.1186/1471-2318-3-4 PMC270050 14594456

[B17] Goldberg M. E. (1991). *The Control of Gaze*. New York: McGraw-Hill

[B18] Graves M. A. Ball K. K. Cissell G. M. West R. E. Whorley K. D. Edwards J. D. (1993). Auditory distraction results in functional visual impairment for some older drivers. *Invest. Ophthalmol. Vis. Sci.* 34 1418–1418

[B19] Hasher L. Stoltzfus E. R. Zacks R. T. Rypma B. (1991). Age and inhibition. *J. Exp. Psychol. Learn. Mem. Cogn.* 17 163–169 10.1037/0278-7393.17.1.163 1826730

[B20] Henderson J. M. (2003). Human gaze control during real-world scene perception. *Trends Cogn. Sci.* 7 498–504 10.1016/j.tics.2003.09.006 14585447

[B21] Henderson J. M. (2006). “Eye movements,” in *Methods in Mind* eds Senior C. Russell T. Gazzaniga M. S. (Cambridge: MIT Press) 171–191

[B22] Hernandez Luna C. P. (2010). *Development and Application of a New Attended Field of View (AFOV) Test*. Master of Science in Vision Science, Waterloo

[B23] Hommel B. Li K. Z. Li S. C. (2004). Visual search across the life span. *Dev. Psychol.* 40 545–558 10.1037/0012-1649.40.4.545 15238042

[B24] Ikeda M. Takeuchi T. (1975). Influence of foveal load on functional visual-field. *Percept. Psychophys.* 18 255–260

[B25] Kanan C. Cottrell G. (2010). Robust classification of objects, faces, and flowers using natural image statistics. *Paper presented at the 2010 IEEE Conference on Computer Vision and Pattern Recognition (CVPR)* San Francisco

[B26] Madden D. J. Gottlob L. R. Allen P. A. (1999). Adult age differences in visual search accuracy: attentional guidance and target detectability. *Psychol. Aging* 14 683–694 10.1037/0882-7974.14.4.683 10632154

[B27] Milleville-Pennel I. Pothier J. Hoc J. M. Mathe J. F. (2010). Consequences of cognitive impairments following traumatic brain injury: pilot study on visual exploration while driving. *Brain Inj.* 24 678–691 10.3109/02699051003692159 20235770

[B28] Muri R. M. Pflugshaupt T. Nyffeler T. Von Wartburg R. Wurtz P. (2005). [A new method of visual exploration analysis]. *Rev. Neurol. (Paris)* 161 513–517 16106802 10.1016/s0035-3787(05)85085-4

[B29] Nasreddine Z. S. Phillips N. A. Bedirian V. Charbonneau S. Whitehead V. Collin I. (2005). The Montreal Cognitive Assessment, MoCA: a brief screening tool for mild cognitive impairment. *J. Am. Geriatr. Soc.* 53 695–699 10.1111/j.1532-5415.2005.53221.x 15817019

[B30] Nef T. Gruber N. Nyffeler T. Müri R. Mosimann U. P. (2013). Development and evaluation of a new instrument to measure visual exploration behavior. *Med. Eng. Phys.* (in press). 10.1016/j.medengphy.2013.09.011 24698394

[B31] Owsley C. Burton-Danner K. Jackson G. R. (2000). Aging and spatial localization during feature search. *Gerontology* 46 300–305 10.1159/000022181 11044783

[B32] Pambakian A. L. Kennard C. (1997). Can visual function be restored in patients with homonymous hemianopia? *Br.J. Ophthalmol.* 81 324–328 9215064 10.1136/bjo.81.4.324 PMC1722157

[B33] Papageorgiou E. Hardiess G. Schaeffel F. Wiethoelter H. Karnath H. O. Mallot H. (2007). Assessment of vision-related quality of life in patients with homonymous visual field defects. *Graefes Arch. Clin. Exp. Ophthalmol.* 245 1749–1758 10.1007/s00417-007-0644-z 17653566

[B34] Pflugshaupt T. Von Wartburg R. Wurtz P. Chaves S. Deruaz A. Nyffeler T. (2009). Linking physiology with behaviour: functional specialisation of the visual field is reflected in gaze patterns during visual search. *Vision Res.* 49 237–248 10.1016/j.visres.2008.10.021 19022277

[B35] Plude D. J. Doussard-Roosevelt J. A. (1989). Aging, selective attention, and feature integration. *Psychol. Aging* 4 98–105 10.1037/0882-7974.4.1.98 2803617

[B36] Plude D. J. Hoyer W. J. (1985). “Attention and performance: identifying and localizing age deficits,” in *Aging and Performance* ed. Charness N. (London: Wiley) 47–99

[B37] Porter G. Tales A. Troscianko T. Wilcock G. Haworth J. Leonards U. (2010). New insights into feature and conjunction search: I. Evidence from pupil size, eye movements and ageing. Cortex 46 621–636 10.1016/j.cortex.2009.04.013 19591979

[B38] Potter L. M. Grealy M. A. Elliott M. A. Andres P. (2012). Aging and performance on an everyday-based visual search task. *Acta Psychol. (Amst.)* 140 208–217 10.1016/j.actpsy.2012.05.001 22664318

[B39] Rabbitt P. (1965). An age-decrement in the ability to ignore irrelevant information. *J. Gerontol. B Psychol. Sci. Soc. Sci.* 20 233–238 10.1093/geronj/20.2.233 14284802

[B40] Robbins K. R. Saxton A. M. Southern L. L. (2006). Estimation of nutrient requirements using broken-line regression analysis. *J. Anim. Sci.* 84 E155–E165 16582088 10.2527/2006.8413_supple155x

[B41] Salthouse T. A. (1993). Speed mediation of adult age-differences in cognition. *Dev. Psychol.* 29 722–738 10.1037//0012-1649.29.4.722

[B42] Schweiger A. Doniger G. M. Dwolatzky T. Jaffe D. Simon E. S. (2003). Reliability of a novel computerized neuropsychological battery for mild cognitive impairment. *Acta Neuropsychol.* 1 407–413

[B43] Scialfa C. T. Esau S. P. Joffe K. M. (1998). Age, target-distractor similarity, and visual search. *Exp. Aging. Res.* 24 337–358 10.1080/036107398244184 9783154

[B44] Scialfa C. T. Joffe K. M. (1997). Age differences in feature and conjunction search: implications for theories of visual search and generalized slowing. *Aging Neuropsychol. Cogn.* 4 227–246 10.1080/13825589708256649

[B45] Scialfa C. T. Kline D. W. Lyman B. J. (1987). Age differences in target identification as a function of retinal location and noise level: examination of the useful field of view. *Psychol. Aging* 2 14–19 10.1037/0882-7974.2.1.14 3268186

[B46] Scialfa C. T. Thomas D. M. Joffe K. M. (1994). Age-differences in the useful field-of-view – an eye-movement analysis. *Optom. Vis. Sci.* 71 736–742 10.1097/00006324-199412000-00003 7898880

[B47] Seiple W. Szlyk J. P. Yang S. Holopigian K. (1996). Age-related functional field losses are not eccentricity dependent. *Vision Res.* 36 1859–1866 10.1016/0042-6989(95)00288-X 8759453

[B48] Sekuler A. B. Bennett P. J. Mamelak M. (2000). Effects of aging on the useful field of view. *Exp. Aging Res.* 26 103–120 10.1080/036107300243588 10755218

[B49] Sekuler R. Ball K. (1986). Visual localization: age and practice. *J. Opt. Soc. Am. A* 3 864–867 10.1364/JOSAA.3.000864 3734925

[B50] Smilek D. Dixon M. J. Merikle P. M. (2006a). Revisiting the category effect: the influence of meaning and search strategy on the efficiency of visual search. *Brain Res.* 1080 73–90 10.1016/j.brainres.2005.07.079 16510131

[B51] Smilek D. Enns J. T. Eastwood J. D. Merikle P. M. (2006b). Relax! Cognitive strategy influences visual search. *Vis. Cogn.* 14 543–564 10.1080/13506280500193487

[B52] Tun P. A. Lachman M. E. (2008). Age differences in reaction time and attention in a national telephone sample of adults: education, sex, and task complexity matter. *Dev. Psychol.* 44 1421–1429 10.1037/a0012845 18793073 PMC2586814

[B53] van de Laar M. C. Van Den Wildenberg W. P. Van Boxtel G. J. Huizenga H. M Van Der Molen M. W. (2012). Lifespan changes in motor activation and inhibition during choice reactions: a Laplacian ERP study. *Biol. Psychol.* 89 323–334 10.1016/j.biopsycho.2011.11.005 22120682

[B54] Wolfe J. M. O’Neill P. Bennett S. C. (1998). Why are there eccentricity effects in visual search? Visual and attentional hypotheses. *Percept. Psychophys.* 60 140–156 10.3758/Bf03211924 9503918

